# Clinical features and hematological and biochemical blood parameters of dogs with hepatobiliary disorders

**DOI:** 10.14202/vetworld.2025.986-993

**Published:** 2025-04-25

**Authors:** Nguyen Vu Thuy Hong Loan, Nguyen Van Chao, Tran Thi Nhung

**Affiliations:** 1 Faculty of Veterinary Medicine and Animal Husbandry, HUTECH University, 475A Dien Bien Phu, Ward 25, Binh Thanh District, Ho Chi Minh City, Vietnam; 2 Department of Veterinary Medicine, Faculty of Animal Sciences and Veterinary Medicine, University of Agriculture and Forestry, Hue University, 102 Phung Hung Street, Hue City, Vietnam

**Keywords:** biochemical analysis, canine hepatology, clinical signs, hematological profile, liver enzymes, Vietnam

## Abstract

**Background and Aim:**

Hepatobiliary disorders represent a significant clinical concern in canine medicine, contributing to substantial morbidity and mortality. However, comprehensive data on clinical presentation and hematological and biochemical alterations associated with these disorders in Vietnam remain limited. This study aimed to characterize the clinical manifestations and hematobiochemical profiles of dogs diagnosed with hepatobiliary disorders in Ho Chi Minh City, Vietnam.

**Materials and Methods:**

Eighty client-owned dogs diagnosed with hepatobiliary disorders through histopathological confirmation were retrospectively evaluated. Clinical signs, age, breed, and sex were recorded. Hematological indices – including red and white blood cell counts, hemoglobin concentration, hematocrit, and platelet (PLT) count – and biochemical parameters – such as total protein (TP), albumin (ALB), globulin, bilirubin, alanine aminotransferase (ALT), aspartate aminotransferase (AST), alkaline phosphatase (ALP), gamma-glutamyl transferase (GGT), blood urea nitrogen (BUN), creatinine (CREA), and uric acid – were assessed. Data were analyzed using one-way analysis of variance and Pearson’s Chi-square test, with statistical significance set at p < 0.05.

**Results:**

Hepatitis was the most prevalent disorder (38.8%), followed by biliary disorders, hepatic lipidosis, neoplastic disorders (each 16.3%), and cirrhosis (12.5%). Vomiting (60.0%), jaundice (57.5%), and diarrhea (48.8%) were among the most frequent clinical signs, with significant variability across disorder types (p < 0.05). A significant variation in PLT counts was observed, with the highest values in hepatic lipidosis cases (p = 0.04). Biochemical analysis revealed marked elevations in total bilirubin, ALT, AST, ALP, and GGT across disorders (p < 0.05), particularly in hepatitis and neoplastic cases. Dogs with cirrhosis exhibited significantly reduced TP and ALB concentrations. Elevated BUN and CREA were also noted in dogs with neoplastic conditions, suggesting concurrent renal involvement.

**Conclusion:**

This study is the first to delineate the clinical and hematobiochemical characteristics of canine hepatobiliary disorders in Vietnam. The findings underscore the diagnostic value of integrating clinical signs with laboratory indices, particularly elevated liver enzymes and hypoalbuminemia, in the identification and differentiation of hepatobiliary conditions. These insights may enhance clinical decision-making and contribute to improved diagnostic accuracy and therapeutic outcomes in veterinary hepatology.

## INTRODUCTION

Hepatobiliary diseases constitute a significant global health concern in small animals, contributing to elevated morbidity and mortality rates. These disorders pose considerable risks to the survival and overall well-being of dogs and cats [1, 2]. Accurate diagnosis necessitates an integrated approach involving clinical evaluation, laboratory testing, histopathology, and cytology [1–4]. In dogs, hepatobiliary disorders often present with diverse and non-specific clinical signs, thereby complicating diagnostic processes. Given the liver’s complex cellular, biliary, and vascular architecture, multiple diagnostic tests have been developed to assess hepatocyte function, membrane integrity, portal circulation, and hepatobiliary and enterohepatic systems [5]. However, due to the intricate interrelations within the liver, diagnostic findings frequently overlap across hepatic, portal, and extrahepatic conditions [6, 7]. In addition, systemic diseases affecting organs such as the lungs, spleen, or kidneys, along with pharmacological interventions, can alter hematological parameters. Consequently, changes in liver enzyme levels may not always indicate primary or secondary hepatic pathology [2]. Although numerous diagnostic methods are available for assessing liver damage and dysfunction, only a few can reliably differentiate between primary liver diseases and secondary hepatic involvement [7, 8].

Dogs with hepatic disorders often exhibit biochemical abnormalities, including alterations in alkaline phosphatase (ALP), aspartate aminotransferase (AST), alanine aminotransferase (ALT), gamma-glutamyl transferase (GGT), cholesterol (low-density lipoprotein and high-density lipoprotein), bile acids, bilirubin, triglycerides, albumin (ALB), and coagulation profiles [8]. Despite their diagnostic value, elevated liver enzyme levels are only moderately correlated with specific disease types, with sensitivity ranging from 60% to 76% [7]. ALT demonstrates the highest sensitivity in detecting hepatocellular necrosis but shows reduced sensitivity in identifying neoplasia (40%) and hepatitis (45%) [9, 10]. ALP elevation is common in cholestatic and parenchymal liver disorders, with sensitivity ranging from 45% to 100%, yet it is significantly less sensitive in acute hepatitis (15%) [9, 11]. Consequently, ALT and ALP levels should be interpreted alongside clinical signs, history, and other diagnostic tests [7, 8]. Hyperlipidemia, characterized by increased triglyceride and/or cholesterol levels, is commonly associated with cholestasis and hepatic insufficiency [12]. Up to 62.2% of dogs with chronic hepatitis exhibit such lipid abnormalities [13], often due to secondary cholestasis [14]. The type and severity of hepatic pathology influence lipid profile alterations [15]. Clinical signs such as ascites [16], hyperbilirubinemia [17, 18], and neutrophilia [19] are indicative of a poor prognosis in chronic hepatitis. Prognostic markers also include hypoalbuminemia, left-shifted leukograms [20], and elevated systemic inflammatory response scores [21].

A prior study by Assawarachan *et al*. [22] in Thailand identified chronic hepatitis (37.9%), liver fibrosis (19.5%), and vacuolar hepatopathy (10.3%) as the most frequent histopathological diagnoses in dogs. Idiopathic chronic hepatitis is histologically characterized by hepatocellular apoptosis or necrosis, hepatic inflammation, and fibrosis [20, 23]. Severe vacuolar hepatopathy can precipitate acute, fulminant hepatic failure [24].

Despite growing global concern regarding canine hepatobiliary disorders, region-specific epidemiological data are lacking – particularly in Vietnam. Previous research has primarily focused on Western and select Asian populations, leaving a gap in the characterization of clinical features, hematological findings, and biochemical profiles in Vietnamese dogs. Moreover, the diagnostic accuracy of liver enzyme abnormalities is hindered by their limited sensitivity and specificity. There remains a paucity of studies correlating clinical signs with laboratory abnormalities, complicating early disease detection. Disparities in diagnostic criteria across studies have further contributed to inconsistencies in classification. The development of standardized diagnostic protocols integrating clinical, hematological, and biochemical markers is therefore warranted. Although several biomarkers have been proposed for prognostication, their reliability in predicting disease progression or therapeutic response is yet to be validated. Breed, age, and sex have been implicated as risk factors in previous studies; however, such data from Vietnamese cohorts are sparse. Identifying these variables could enhance prevention and early detection. Furthermore, most existing studies are cross-sectional, providing only a snapshot of disease states. Longitudinal studies tracking disease evolution, therapeutic response, and survival are essential for optimizing clinical management. Addressing these research gaps will improve diagnostic precision, therapeutic strategies, and clinical outcomes in canine hepatobiliary disorders.

Given the limited data on hepatobiliary disorders in canine populations in Vietnam and the diagnostic challenges posed by the non-specific clinical and laboratory manifestations of these conditions, this study aimed to characterize the clinical features and evaluate the hematological and biochemical blood parameters of dogs diagnosed with hepatobiliary disorders. By examining variations across different histopathological categories, the study sought to identify key diagnostic indicators that may aid in improving the accuracy of disease recognition, prognosis, and treatment strategies in veterinary clinical practice.

## MATERIALS AND METHODS

### Ethical approval

This study was based on processing of the samples received from different small animal hospitals so, ethical approval was not necessary for this study. However, samples from all small animal hospitals were collected by a trained veterinarian/veterinary nurse.

### Study period and location

The study was conducted from January to Decembe**r** 2023. The samples were collected from several small animal hospitals in Ho Chi Minh City, Vietnam. The samples were processed at Nong Lam University Veterinary Hospital (Quarter 5, Linh Trung Ward, Thu Duc City, Ho Chi Minh City).

### Data collection and study population

A total of 80 canine patients of both sexes were enrolled in this study, which exhibited clinical signs suggestive of hepatobiliary disorders. All cases were confirmed as hepatobiliary disorders through histopathological diagnosis. Each dog underwent a thorough clinical assessment, which included an evaluation of general condition, mucous membranes, hydration status, pain response, and abdominal distension. The collected data encompassed breed, age, sex, and clinical symptoms such as vomiting, jaundice, diarrhea, abnormal urine coloration, polydipsia, debilitation, and neurological signs. Cases lacking complete information on breed, age, or sex were excluded from the study. Furthermore, dogs with histopathological findings unrelated to hepatobiliary disease were also omitted from the study.

### Histopathological evaluation

Certified anatomical pathologists conducted histopathological examinations at multiple veterinary diagnostic laboratories affiliated with small animal hospitals in Ho Chi Minh City. Based on the World Small Animal Veterinary Association Standards [25], cases were categorized into five major morphological types: Cirrhosis, biliary disorders, hepatitis, hepatic lipidosis, and neoplastic disorders.

### Hematological analysis

Approximately 2 mL of whole blood was drawn from the cephalic vein into ethylenediaminetetraacetic acid-treated tubes (MEd Comtech, Ho Chi Minh City, Vietnam) for hematological analysis. Samples were immediately processed using an automated hematology analyzer (Mindray BC-20, ICEN Technology Company Limited, China) to measure red blood cell (RBC) count, hemoglobin (HGB) concentration, hematocrit (HCT), white blood cell (WBC) count, and platelet (PLT) count.

### Biochemical analysis

For biochemical assessment, 2 mL of blood was collected into plain serum tubes. After clotting, samples were centrifuged, and the serum was transferred to sterile Eppendorf tubes (Thermo Scientific Hampshire, UK). The following biochemical parameters were analyzed using the Cobas 6000 analyzer (Roche, F. Hoffmann-La Roche Ltd., Basel, Switzerland): Total protein (TP), ALB, globulin (GLB), total bilirubin (T-BIL), ALT, AST, ALP, GGT, blood urea nitrogen (BUN), creatinine (CREA), and uric acid (UA).

### Statistical analysis

Statistical analyses were performed using the Statistical Package for the Social Sciences version 18.0 (IBM, Armonk, NY, USA). Continuous variables, including hematological and biochemical parameters, were tested for normal distribution and analyzed using one-way analysis of variance with *post hoc* tests. Data were expressed as mean ± standard deviation. Categorical variables such as sex, breed, age, and clinical signs were presented as counts and percentages and analyzed using Pearson’s chi-square test. p < 0.05 was considered statistically significant.

## RESULTS

A total of 80 dogs diagnosed with hepatobiliary disorders were included in this study. Among the various conditions identified, hepatitis was the most prevalent (38.8%, 31/80), followed by biliary disorders, hepatic lipidosis, and neoplastic disorders (each accounting for 16.3%, 13/80), and cirrhosis (12.5%, 10/80). The study population consisted of 45 female dogs (56.3%) and 35 male dogs (43.8%). No statistically significant differences were observed in the distribution of sex, age, or breed among dogs with different types of hepatobiliary disorders (p > 0.05) (Table 1).

**Table 1:** Distribution of hepatobiliary disorders according to age, breed, and sex of dog, n (%).

Parameters	Total	Cirrhosis	Biliary	Hepatitis	Hepatic lipidosis	Neoplastic disorders
N	80	10 (12.5)	13 (16.3)	31 (38.8)	13 (16.3)	13 (16.3)
Age						
<5 years	14 (17.5)	0	3 (21.4)	10 (71.4)	1 (7.1)	0
6–10 years	37 (46.3)	5 (13.5)	4 (10.8)	13 (35.1)	8 (21.6)	7 (18.9)
>10 years	29 (36.3)	5 (17.2)	6 (20.7)	8 (27.6)	4 (13.8)	6 (20.7)
Breed						
Vietnamese dog	45 (56.3)	8 (17.8)	5 (11.1)	17 (37.8)	5 (11.1)	10 (22.2)
Mixed breed	35 (43.8)	2 (5.7)	8 (22.9)	14 (40.0)	8 (22.9)	3 (8.6)
Gender						
Female	45 (56.3)	7 (15.6)	7 (15.6)	17 (37.8)	6 (13.3)	8 (17.8)
Male	35 (43.8)	3 (8.6)	6 (17.1)	14 (40.0)	7 (20.0)	5 (14.3)

All enrolled dogs exhibited at least two to six clinical signs associated with hepatobiliary dysfunction. These signs included vomiting (60.0%, 48/80), jaundice (57.5%, 46/80), diarrhea and abnormal urine coloration (each 48.8%, 39/80), polydipsia (46.3%, 37/80), debilitation (42.5%, 34/80), ascites (41.3%, 33/80), and neurological signs (21.3%, 17/80) (Table 2). Statistically significant differences in clinical presentation were found among the different disorder groups (p < 0.05). Vomiting and diarrhea were most frequently observed in dogs with hepatic lipidosis (69.2%, 9/13), followed by those with hepatitis (67.7%, 21/31; 54.8%, 17/31), biliary disorders (61.5%, 8/13; 53.8%, 7/13), neoplastic disorders (53.8%, 7/13; 30.8%, 4/13), and cirrhosis (30.0%, 3/10; 20.0%, 2/10). The incidence of jaundice was highest in dogs with biliary disorders (76.9%, 10/13), neoplastic disorders (69.2%, 9/13), hepatitis (67.7%, 21/31), and cirrhosis (50.0%, 5/10), but was least frequent in dogs with hepatic lipidosis (7.7%, 1/13).

**Table 2:** Clinical signs in dogs with different hepatobiliary disorders, n (%).

Cinical signs	Total	Cirrhosis	Biliary	Hepatitis	Hepatic lipidosis	Neoplastic disorders
Total	80	10 (12.5)	13 (16.3)	31 (38.8)	13 (16.3)	13 (16.3)
Diarrhea	39 (48.8)	2 (20.0)^a^	7 (53.8)^abc^	17 (54.8)^bc^	9 (69.2)^c^	4 (30.8)^ab^
Debilitating	34 (42.5)	9 (90.0)^a^	3 (23.1)^b^	11 (35.5)^b^	3 (23.1)^b^	8 (61.5)^ac^
Vomiting	48 (60.0)	3 (30.0)^a^	8 (61.5)^ab^	21 (67.7)^b^	9 (69.2)^ab^	7 (53.8)^ab^
Polydipsia	37 (46.3)	6 (60.0)	5 (38.5)	16 (51.6)	6 (46.2)	4 (30.8)
Abnormally colored urine	39 (48.8)	3 (30.0)^ab^	9 (69.2)^a^	19 (61.3)^a^	1 (7.7)^b^	7 (53.8)^a^
Ascites	33 (41.3)	6 (60.0)^ab^	3 (23.1)^a^	10 (32.3)^a^	9 (68.2)^b^	5 (38.5)^ab^
Jaundice	46 (57.5)	5 (50.0)^a^	10 (76.9)^a^	21 (67.7)^a^	1 (7.7)^b^	9 (69.2)^a^
Nervous signs	17 (21.3)	3 (30.0)^ab^	1 (7.7)^a^	5 (16.1)^a^	1 (7.7)^a^	7 (53.8)^b^

^a,b,c^Variables with different superscripts in the same row are significantly different at p *<* 0.05.

Hematological findings are summarized in Table 3. A statistically significant difference was noted in PLT counts among the different hepatobiliary conditions (standard error of the mean = 16.6 × 10^9^/L, p = 0.04). However, there were no significant differences in WBC, RBC, HGB, or HCT levels (p > 0.05). The average WBC, RBC, HGB, HCT, and PLT values across all cases were 24.9 × 10^9^/L, 6.3 × 10^12^/L, 127.3 g/L, 41.9%, and 246.1 × 10^9^/L, respectively, with WBC and RBC levels exceeding the normal reference range. The highest PLT count was observed in dogs with hepatic lipidosis (346.3 × 10^9^/L), followed by those with hepatitis (250.3 × 10^9^/L), cirrhosis (233.6 × 10^9^/L), neoplastic disorders (219.0 × 10^9^/L), and biliary disorders (171.7 × 10^9^/L). In addition, dogs with biliary disorders exhibited lower HGB concentrations (103.6 g/L) compared to those with hepatic lipidosis (164.9 g/L).

**Table 3:** Hematological blood parameters (mean ± SD) of dogs with hepatobiliary disorders.

Parameters	Total	Reference range	Cirrhosis	Biliary	Hepatitis	Hepatic lipidosis	Neoplastic disorders	SEM	p-values
WBC (×10^9^/L)	24.96 ± 17.4	6–17	16.6 ± 6.9	29.1 ± 10.8	29.7 ± 21.7	16.9 ± 12.8	23.9 ± 16.8	1.9	0.08
RBC (×10^12^/L)	6.3 ± 2.6	5.5–8.5	5.9 ± 2.5	5.6 ± 2.2	6.0 ± 2.8	7.7 ± 1.2	6.2 ± 3.1	0.3	0.25
HGB (g/L)	127.3 ± 55.9	110–190	126.0^ab^ ± 57.2	103.6^a^ ± 58.2	121.5^ab^ ± 55.9	164.9^b^ ± 23.7	128.3^ab^ ± 64.6	6.3	0.07
HCT (%)	41.9 ± 14.4	39–56	38.0 ± 15.3	38.4 ± 14.8	41.0 ± 15.1	50.9 ± 7.2	41.7 ± 15.0	1.6	0.15
PLT (×10^9^/L)	246.1 ± 148.5	117–460	233.6^ab^ ± 152.6	172.7^a^ ± 138.8	250.3^a^ ± 155.9	346.3^b^ ± 82.2	219.0^a^ ± 152.5	16.6	0.04

WBC=White blood cell, RBC=Read blood cell, HGB=Hemoglobin, HCT=Hematocrit, PLT=Platelet count, SEM=Standard error of the mean, SD=Standard deviation. ^a,b,c^Variables with different superscripts in the same row are significantly different at p *<* 0.05.

Biochemical analyses provided further insights (Table 4). Elevated levels were recorded for T-BIL (1.7 mg/dL), ALT (326.4 U/L), AST (111.6 U/L), ALP (634.1 U/L), GGT (27.4 U/L), BUN (35.3 mg/dL), and CREA (2.0 mg/dL), all above the reference ranges. The mean values for TP, ALB, ALT, AST, ALP, and GGT were 6.2 g/dL, 2.4 g/dL, 316.4 U/L, 111.6 U/L, 634.1 U/L, and 27.4 U/L, respectively, with significant differences observed among disorder types (p < 0.05). Dogs with hepatitis had the highest mean ALT (545.9 U/L) and AST (176.8 U/L) levels. The lowest TP concentration was observed in dogs with cirrhosis (4.6 g/dL), compared to higher values in biliary disorders (6.2 g/dL), hepatitis (6.2 g/dL), hepatic lipidosis (7.2 g/dL), and neoplastic disorders (6.4 g/dL) (p < 0.05). Although the mean values for GLB, T-BIL, BUN, CREA, and UA did not differ significantly overall (p > 0.05), pairwise comparisons revealed significantly higher GLB levels in neoplastic cases (4.5 g/dL) than in cirrhosis (1.4 g/dL) and significantly elevated BUN in neoplastic disorders (60.0 mg/dL) compared to biliary disorders (21.6 mg/dL) (p < 0.05).

**Table 4:** Biochemical blood parameters (mean ± SD) of canine patients with hepatobiliary disorders.

Parameters	Total	Reference range	Cirrhosis	Biliary	Hepatitis	Hepatic lipidosis	Neoplastic disorders	SEM	p-values
TP (g/dL)	6.2 ± 1.9	5.2–8.2	4.6^a^ ± 1.6	6.2^b^ ± 2.3	6.2^b^ ± 1.9	7.2^b^ ± 1.1	6.4^b^ ± 1.8	0.2	0.03
ALB (g/dL)	2.4 ± 0.9	2.3–4.4	1.4^a^ ± 0.6	2.3^b^ ± 0.6	2.5c ± 0.8	3.2c ± 0.6	1.9^b^ ± 0.8	0.1	<0.001
GLB (g/dL)	3.8 ± 1.7	2.3–5.2	3.1^a^ ± 1.6	3.9^ab^ ± 2.1	3.7^ab^ ± 1.8	3.9^ab^ ± 1.2	4.5^b^ ± 1.7	0.2	0.42
T-BIL (mg/dL)	1.7 ± 1.9	0–0.9	1.0^ab^ ± 1.0	1.9^ab^ ± 1.3	2.1^a^ ± 2.6	0.5^b^ ± 0.5	2.2^a^ ± 1.4	0.2	0.07
ALT (U/L)	326.4 ± 321.9	0–112	146.4^a^ ± 156.3	290.3^a^ ± 203.7	545.9^b^ ± 367.0	80.4^a^ ± 55.2	223.4^a^ ± 229.1	36.0	<0.001
AST (U/L)	111.6 ± 108.0	0–50	68.6^a^ ± 64.5	95.9^a^ ± 97.8	176.8^b^ ± 120.0	36.9^a^ ± 18.2	79.5^a^ ± 88.2	12.1	<0.001
ALP (U/L)	634.1 ± 753.9	10–112	274.2^ab^ ± 268.2	877.3^a^ ± 536.2	829.2^ab^ ± 893.6	201.7^b^ ± 189.8	634.8^ab^ ± 945.9	84.3	0.04
GGT (U/L)	27.4 ± 28.0	0–11	13.5^a^ ± 13.6	24.5^ab^ ± 15.9	39.8^b^ ± 36.4	19.1^ab^ ± 20.9	19.6^ab^ ± 17.9	3.1	0.03
BUN (mg/dL)	35.3 ± 36.9	7–28.2	27.7^ab^ ± 22.7	21.6^a^ ± 13.4	34.4^ab^ ± 40.1	32.2^ab^ ± 32.9	60.0^b^ ± 48.8	4.1	0.08
CREA (mg/dL)	2.0 ± 3.9	0.3–1.5	0.9^ab^ ± 1.3	0.7^a^ ± 0.3	2.1^ab^ ± 4.3	1.1^ab^ ± 0.9	5.5^b^ ± 6.4	0.4	0.07
UA (mg/dL)	0.1 ± 0.45	0–1	0.04 ± 0.02	0.05 ± 0.03	0.13 ± 0.32	0.07 ± 0.04	0.32 ± 1.01	0.1	0.52

TP=Total protein, ALB=Albumin, GLB=Globulin, T-BIL=Total bilirubin, ALT=Alanine aminotransferase, AST=Aspartate aminotransferase, ALP=Alkaline phosphatase, GGT=Gamma-glutamyl transferase, BUN=Blood urea nitrogen, CREA=Creatinine, UA=Uric acid, SEM=Standard error of the mean, SD=Standard deviation. ^a,b,c^Variables with different superscript in the same row are significantly different at p *<* 0.05

## DISCUSSION

In recent years, hepatobiliary disorders have emerged as a significant cause of increased morbidity and mortality in dogs. Although elevated liver enzyme levels are important initial indicators for diagnosing these disorders, their limited sensitivity underscores the necessity for more specific biomarkers. This is the first study to describe the clinical characteristics and hematobiochemical parameters associated with hepatobiliary diseases in dogs in Vietnam (Table 1). Hepatitis was identified as the most common hepatobiliary disorder, aligning with the findings reported by Bandara *et al*. [26]. Conversely, a study by Hirose *et al*. [27] in Japan indicated that vascular disorders were the most prevalent, with hepatitis ranking second. Prior studies by Bandara *et al*. [26] and Hirose *et al*. [27] have shown that the distribution of hepatobiliary disorders in dogs can be influenced by factors such as age, breed, and sex; however, the findings of the present investigation do not fully conform to those previously reported.

Clinical manifestations such as diarrhea, debilitation, vomiting, discolored urine, ascites, jaundice, and neurological signs were commonly observed in the affected dogs (Tables 2–4). These findings are consistent with those of Phosri *et al*. [28], who reported vomiting, diarrhea, polydipsia, ascites, and jaundice as common symptoms. Nevertheless, clinical presentations can be highly variable, ranging from anorexia and weight loss to abdominal effusion and respiratory distress [29]. In the present study, ascites and jaundice were among the most frequently noted signs. Other less common clinical features included anemia, weight gain, limb edema, and lethargy [2].

Diarrhea in dogs with hepatobiliary disease may result from reduced bile secretion into the duodenum, impairing fat absorption, or from portal hypertension and vascular congestion, which enhance intestinal fluid accumulation [25]. The vomiting observed may be attributed to impaired hepatic clearance of endotoxins, which stimulates the vomiting center in the brain’s fourth ventricle [30]. Jaundice arises due to bilirubin accumulation, normally excreted by the liver following RBC breakdown, when hepatic function is compromised. This results in visible yellowing of the skin, mucous membranes, sclerae, and pinnae [31, 32]. Correspondingly, elevated T-BIL levels were observed in the dogs evaluated in this study (Table 4). Ascites was documented in 33 out of 80 dogs (41.3%), most commonly in those with cirrhosis and hepatitis, likely due to increased portal venous pressure [7]. ALB, synthesized exclusively by the liver, is the main contributor to plasma oncotic pressure. Hence, hypoalbuminemia, commonly seen in hepatobiliary disorders, likely contributed to the development of ascites in the studied dogs [33].

A significant reduction in PLT counts was also observed, consistent with reports by Nantasanti *et al*. [1] and Tantary *et al*. [34]. Possible mechanisms for thrombocytopenia in such cases include splenic sequestration due to congestion, reduced hepatic thrombopoietin synthesis, immune-mediated PLT destruction [35], and increased PLT consumption caused by hemorrhage or vascular compromise. In addition, impaired hepatic synthesis of clotting factors may lead to prolonged coagulation times. Webster [36] emphasized that the liver serves as the primary site for coagulation factor production, and its dysfunction results in clinically evident coagulopathies [35].

Marked elevations in TP, T-BIL, AST, ALT, ALP, and GGT were recorded among dogs with hepatobiliary disorders. Increased serum transaminases, including ALT, AST, and GGT, reflect hepatocyte membrane injury, hepatic inflammation, and necrosis, with the magnitude of elevation correlating with the extent of hepatocellular damage [13, 21]. ALP, a membrane-bound enzyme located on hepatic tubules and biliary epithelial cells, was also significantly elevated. Lakshmi *et al*. [37] similarly reported heightened ALP and GGT activity in cases of cirrhosis, hepatitis, cholestasis, and gallstones. The elevated T-BIL levels observed further corroborate the presence of hyperbilirubinemia, which results from disrupted bilirubin metabolism and excretion due to hepatocellular injury or biliary obstruction [17, 38].

In addition, this study documented significant reductions in serum ALB and GLB levels in affected dogs (Table 4). Since the liver is responsible for the synthesis and degradation of most plasma proteins, conditions such as hepatitis and cirrhosis commonly result in hypoalbuminemia. This decrease may also stem from reduced dietary intake, malabsorption, vomiting, or diarrhea [22, 29]. These observations are in agreement with previous findings by Kozat and Sepehrizadeh [7] and Sevelius [11]. However, hypoalbuminemia may not solely reflect decreased hepatic synthesis; it can also result from ALB leakage into the hepatic lymph or an expanded distribution volume, particularly in ascitic patients. Furthermore, serum CREA levels were significantly elevated, corroborating findings by Prbavathy *et al*. [38], and possibly indicating concurrent renal involvement or impaired renal clearance.

## CONCLUSION

This study provides the first comprehensive clinical and hematobiochemical characterization of hepatobiliary disorders in dogs in Vietnam. Among the evaluated cases, hepatitis emerged as the most prevalent disorder, followed by biliary disorders, hepatic lipidosis, neoplastic diseases, and cirrhosis. Key clinical signs, including vomiting, jaundice, diarrhea, and ascites were significantly associated with specific hepatobiliary pathologies. Hematological analysis revealed significant alterations in PLT counts, while biochemical assessments showed notable increases in liver enzymes (ALT, AST, ALP, and GGT), T-BIL, and urea concentrations, particularly in dogs diagnosed with hepatitis and neoplasia. These findings underscore the diagnostic value of integrating clinical symptoms with hematological and biochemical indices to improve disease classification and management.

A major strength of this study lies in its use of histopathological confirmation to classify hepatobiliary disorders, enhancing diagnostic accuracy. Furthermore, the inclusion of a well-defined clinical population and the application of standardized laboratory methods lend reliability to the findings.

However, this study is subject to several limitations. First, the sample size, although adequate for preliminary analysis, may limit the generalizability of the findings across broader canine populations. Second, the cross-sectional design precludes assessment of disease progression, response to therapy, or long-term outcomes. In addition, certain potential confounders, such as concurrent diseases or nutritional status were not fully explored, which could influence biochemical profiles.

Future research should prioritize longitudinal cohort studies to monitor the progression of hepatobiliary diseases and treatment efficacy over time. Expanding the sample size and including multi-center data would further validate these findings. In addition, molecular and genetic profiling could help identify breed-specific susceptibilities or biomarker candidates for early detection and prognostication.

This study offers foundational data on the clinical and laboratory profiles of hepatobiliary disorders in Vietnamese dogs and provides a framework for improving diagnostic strategies and guiding future investigative efforts in veterinary hepatology.

## AUTHORS’ CONTRIBUTIONS

NVTHL, NVC, and TTN: Study conception and design, interpretation of the results, and drafted and revised the manuscript. NVC and NVTHL: Developed the original hypothesis and designed the study. NVTHL and TTN: Data collection and analysis. All authors have read and approved the final manuscript.
